# Does the development of new medicinal products in the European Union address global and regional health concerns?

**DOI:** 10.1186/1478-7954-8-34

**Published:** 2010-12-20

**Authors:** Ferrán Catalá-López,, Anna García-Altés,, Elena Álvarez-Martín, Ricard Gènova-Maleras, Consuelo Morant-Ginestar

**Affiliations:** 1 Fundación Instituto de Investigación en Servicios de Salud, Valencia, Spain; 2 Division of Pharmacoepidemiology and Pharmacovigilance, Spanish Medicines and Healthcare Products Agency (AEMPS), Ministry of Health and Social Policy, Madrid, Spain; 3 Catalan Agency for Health Information, Assessment and Quality (CAHIAQ), Barcelona, Spain; 4 Department of Preventive Medicine and Public Health, Rey Juan Carlos University, Madrid, Spain; 5 Primary Care General Directorate, Regional Health Council, Madrid, Spain; 6 Department of Health Information Systems, Regional Health Council, Madrid, Spain

## Abstract

**Background:**

Since 1995, approval for many new medicinal products has been obtained through a centralized procedure in the European Union. In recent years, the use of summary measures of population health has become widespread. We investigated whether efforts to develop innovative medicines are focusing on the most relevant conditions from a global public health perspective.

**Methods:**

We reviewed the information on new medicinal products approved by centralized procedure from 1995 to 2009, information that is available to the public in the European Commission Register of medicinal products and the European Public Assessment Reports from the European Medicines Agency. Morbidity and mortality data were included for each disease group, according to the Global Burden of Disease project. We evaluated the association between authorized medicinal products and burden of disease measures based on disability-adjusted life years (DALYs) in the European Union and worldwide.

**Results:**

We considered 520 marketing authorizations for medicinal products and 338 active ingredients. New authorizations were seen to increase over the period analyzed. There was a positive, high correlation between DALYs and new medicinal product development (ρ = 0.619, p = 0.005) in the European Union, and a moderate correlation for middle-low-income countries (ρ = 0.497, p = 0.030) and worldwide (ρ = 0.490, p = 0.033). The most neglected conditions at the European level (based on their attributable health losses) were neuropsychiatric diseases, cardiovascular diseases, respiratory diseases, sense organ conditions, and digestive diseases, while globally, they were perinatal conditions, respiratory infections, sense organ conditions, respiratory diseases, and digestive diseases.

**Conclusions:**

We find that the development of new medicinal products is higher for some diseases than others. Pharmaceutical industry leaders and policymakers are invited to consider the implications of this imbalance by establishing work plans that allow for the setting of future priorities from a public health perspective.

## Background

Medicinal product policies are a challenging field in current health policy. Medicines are a major determining factor of population health [1], as the current capacity of public health to prevent, discontinue, or modify the natural course of diseases and reduce their symptoms depends mostly on them.

The pharmaceutical industry is one of the most potent industrial sectors in developed countries, having a major impact on national economies and the creation of employment opportunities as well as research investment policies. A proxy variable to establish pharmaceutical research success is the number of authorizations granted for marketing medicinal products. Regulatory authorities generally judge the suitability of new treatments in terms of the benefit-risk balance and regulate commercialization and conditions of use based on quality, safety, and efficacy criteria. The benefit of medicinal products (if they do not harm) can be viewed as the decrease in disease burden associated with their use [2], though additional criteria also can be considered (e.g., degree of innovation, cost-effectiveness, added therapeutic value, existence of medicinal products or other options for the same conditions, and specific needs of some population groups).

Since early 1995, European Union (EU) authorization for many new human-use medicinal products has been obtained through a centralized procedure. This procedure, managed by the European Medicines Agency, is mandatory for products derived from biotechnology and other high-technology procedures, those aimed at the treatment of human immunodeficiency virus/acquired immunodeficiency syndrome (HIV/AIDS), cancer, diabetes, neurodegenerative diseases, autoimmune disorders, viral diseases, and also orphan medicines used to treat rare diseases. The centralized authorization application also can be submitted whenever the medicinal product involved is a major therapeutic, scientific, or technical innovation, or is relevant in any other way for population health [3].

The existence of limited resources for research and development activities, together with budget restrictions for funding approved medicinal products, require establishing explicit criteria to guide debates on priority-setting. In recent years, the calculation and use of summary measures of population health have become widespread and influential in global health, as in the case of disability-adjusted life years (DALYs) [4]. DALYs for a disease or health condition are calculated as the sum of years of life lost (YLL) due to premature mortality in relation to life expectancy and years lived with disability (YLD). One DALY is equivalent to the loss of one year of healthy life. DALYs allow the burden of disease in a population to be measured as the gap between current health and an ideal situation where everyone lives to old age, free of disease and disability. It is an indication of the unfinished health research agenda and identifies areas where health gains can be made [5]. Thus, DALYs are designed to guide investment health policies and to inform global priority setting for health research activities [6,7].

The aim of this paper is to examine whether medicines development activities in the EU are aimed at the most relevant conditions from a global public health perspective, considering burden of disease criteria.

## Methods

### Study design and data sources

This is an observational, cross-sectional study evaluating the association between burden of disease measures and human-use medicinal products authorized in the EU, establishing whether the development of innovative medicinal products changes with disease burden in different populations. With information from public sources [8,9] accessible through the Internet, we created an authorization database based on the cohort of medicinal products authorized by EU centralized procedure during the period from January 1995 (the date when the European Medicines Agency was created) to December 2009. We specifically examined: the European Commission Register of medicinal products [8] and the European Public Assessment Reports (EPARs) for authorized medicinal products [9]. We collected the following information: year of approval, name of the medicinal product, active ingredients (new chemical entities), main indication, disease seriousness, type of intervention, and other variables when appropriate (e.g., new mechanism, biotechnological/biological product, orphan medicinal product, fixed-dose combinations or drug associations, generics and similar biological medicinal products - also called "biosimilars").

We used the following definitions:

Main indication: This was defined as the first approval for a given drug, and later approvals were considered extensions.

Disease seriousness: A disease was classified as (chronic) serious if it is fatal, requires hospitalization, or is life-threatening or heavily disabling (e.g., myocardial infarction, dementia, pneumonia); as nonserious (e.g., erectile dysfunction, allergic rhinitis); or as a risk factor for serious disease (e.g., obesity, hypertension, anemia associated with chronic renal failure).

Type of intervention: This was defined as prevention, diagnosis, treatment, or palliative care/rehabilitation.

New mechanism of action: A mechanism was defined as new if the primary pharmacodynamic target (e.g., receptor) and drug-target interaction differed from existing drugs. When a medicine belonged to a new therapeutic class and the mechanism of action was unknown, it was classified as new. 

Medicinal products for which marketing authorization was "withdrawn, suspended and/or not renewed" during the study period were excluded from the primary analysis.

The Pediatric Regulation (Regulation EC No 1901/2006) came into place to improve the health of children in the EU without subjecting children to unnecessary trials or delaying the authorization of medicinal products for use in adults. Since then, all patented medicinal products submitted for a marketing authorization (or line extension) in the EU need to have an agreed drug development plan, namely a Pediatric Investigation Plan (PIP). Thus, to create a complementary assessment of medicines development activities related to pediatric needs, the European Medicines Agency website http://www.ema.europa.eu was searched for information with respect to opinions on submitted PIPs (proxy variable used for pediatric pharmaceutical output). We also included decisions agreeing on a PIP, with or without partial waiver(s) and or deferral(s). These data provide information about what medicinal products are in the pipeline and may help illuminate the extent to which efforts are focusing on the development of innovative medicines for children.

For each disease group, we included morbidity and mortality data according to the Global Burden of Disease study [10,11]. This project was conducted by the World Bank, the World Health Organization (WHO), and the Harvard School of Public Health to quantify the burden of disease and injury on human populations and to gather information on prevalence, incidence, severity, disability, and mortality for more than 100 causes [4,10]. Burden of disease, measured in DALYs and mortality, refers to the most recent estimations available at the time of analysis. In brief, this report used the data for the world population - by income categories as defined by the World Bank - and for the 25 EU Member States (EU-25) in 2004 [11]. The main therapeutic indications for medicinal products were then matched with the categories of the disease classification system defined in the Global Burden of Disease study [10].

### Statistical analysis

We established the direction and strength of association between human-use medicinal products authorized during the study period and burden of disease measures. In a subanalysis regarding pharmaceutical development output for pediatric needs, we measured the associations between the burden of disease in children under 15 years of age - the pediatric age group reported in the estimates for WHO countries [10,11] - and the decisions of the European Medicines Agency's Pediatric Committee agreeing on a PIP for an individual medicinal product. Using the STATA package (Version 10, StataCorp LP, College Station, TX, USA), we calculated nonparametric Spearman rank correlation coefficients (ρ). The statistical significance level was set at a value of 0.05 (a confidence level of 95%).

## Results

**Figure 1:**
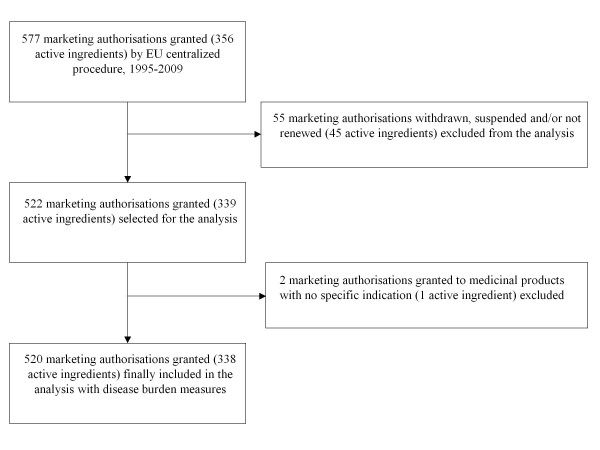
**Pharmaceutical output selection. Marketing authorizations granted (and active ingredients) by EU centralized procedure, 1995-2009**.

We included 520 marketing authorizations for medicinal products in the analysis, corresponding to 338 active ingredients (Figure 1). Table 1 describes the main characteristics and distribution of the new medicinal products in development. From 1995 to 2009, the total number of new authorizations by EU centralized procedure increased. Furthermore, the ratio between the number of marketing authorizations and the number of active ingredients also increased over the period of study. We found that medicinal products covered mainly therapeutic and preventive interventions aimed at serious diseases.

**Table 1:** Main characteristics and distribution of the marketing authorizations of medicinal products for human use and active ingredients (NCEs), 1995-2009

	Period 1995-1999		Period 2000-2004		Period 2005-2009	
**Characteristics**	**Num. of authorizations (%)**	**Num. of NCEs (%)**	**Num. of authorizations (%)**	**Num. of NCEs (%)**	**Num. of authorizations (%)**	**Num. of NCEs (%)**

**Total**	**86 (100)**	**70 (100)**	**143 (100)**	**116 (100)**	**291 (100)**	**152 (100)**

**Type of intervention**						
Prevention	15 (17.4)	12 (17.1)	13 (9.1)	7 (6.0)	56 (19.2)	19 (12.5)
Diagnosis	4 (4.6)	3 (4.3)	4 (2.8)	4 (3.4)	5 (1.7)	5 (3.3)
Treatment	65 (75.6)	53 (75.7)	122 (85.3)	101 (87.1)	214 (73.5)	120 (78.9)
Palliative care/rehabilitation	2 (2.3)	2 (2.9)	4 (2.8)	4 (3.4)	16 (5.5)	8 (5.3)

**Disease seriousness**						
Serious disease	59 (68.6)	50 (71.4)	104 (72.7)	86 (74.1)	191 (65.6)	109 (71.7)
Nonserious disease	11 (12.8)	9 (12.9)	21 (14.7)	17 (14.6)	44 (15.1)	20 (13.2)
Risk factor for serious disease	16 (18.6)	11 (15.7)	18 (12.6)	13 (11.2)	56 (19.2)	23 (15.1)

**New mechanism of action**	**18 (20.9)**	**17 (24.3)**	**44 (30.8)**	**42 (36.2)**	**32 (11.0)**	**27 (17.8)**

**Fixed-dose combinations**	**6 (7.0)**	**4 (5.7)**	**13 (9.1)**	**8 (6.9)**	**37 (12.7)**	**18 (11.8)**

**Biotechnological/biological product**	**27 (31.4)**	**24 (34.3)**	**52 (36.4)**	**41 (35.3)**	**77 (26.5)**	**42 (27.6)**

Orphan medicinal product*	-	-	**18 (12.6)**	**18 (15.5)**	**39 (13.4)**	**39 (25.7)**

Generic and/or "biosimilar"*	-	-	-	-	**69 (23.7)**	-

Data do not sum to 100% because of rounding errors *The European Commission granted marketing authorizations for: orphan medicinal products in 2001, "biosimilar" products in 2006, and generic products in 2007. NCEs = New chemical entities.

Table 2 shows the main data on the output of pharmaceutical development and disease burden measures by cause and region. The indication of new active ingredients was mainly malignant neoplasms (17.5%), infectious and parasitic diseases (16.6%), blood and endocrine disorders (12.7%), neuropsychiatric conditions (10.9%), cardiovascular diseases (8.6%), diabetes mellitus (7.4%), and musculoskeletal diseases (7.1%).

**Table 2:** Distribution of medicinal products authorized for human use by therapeutic area (1995-2009) and burden of disease (as measured by disability-adjusted life years and mortality)

Disease conditions	Medicinal products for human use, 1995-2009		Burden of disease in the population			
	**Marketing authorizations**	**NCEs**	**Mortality per 10**^**6 **^**(%)**		**DALYs per 10**^**6 **^**(%)**	
	**Number (%)**	**Number (%)**	**Worldwide**	**EU-25**	**Worldwide**	**EU-25**

**Communicable diseases**	**100 (19.2)**	**75 (22.2)**	**18.0 (30.6)**	**0.2 (4.8)**	**604.0 (39.7)**	**2.7 (4.6)**
Infectious and parasitic diseases	68 (13.1)	56 (16.6)	9.5 (16.2)	.06 (1.3)	302.1 (19.8)	0.9 (1.6)
Respiratory infections	19 (3.6)	8 (2.4)	4.3 (7.2)	0.1 (3.1)	97.8 (6.4)	0.6 (1.0)
Maternal conditions	11 (2.1)	9 (2.7)	0.5 (0.9)	.00 (0.0)	38.9 (2.6)	0.2 (0.3)
Perinatal conditions	2 (0.4)	2 (0.6)	3.2 (5.4)	.01 (0.3)	126.4 (8.3)	0.6 (1.0)

**Noncommunicable diseases**	**419 (80.6)**	**262 (77.5)**	**35.0 (59.6)**	**4.0 (89.9)**	**731.6 (48.0)**	**51.1 (87.4)**
Malignant neoplasms	73 (14.0)	59 (17.5)	7.4 (12.6)	1.2 (27.0)	77.8 (5.1)	9.7 (16.6)
Other neoplasms	1 (0.2)	1 (0.3)	0.2 (0.3)	.03 (0.8)	1.9 (0.1)	0.2 (0.3)
Diabetes mellitus	46 (8.8)	25 (7.4)	1.1 (1.9)	0.1 (2.3)	19.7 (1.3)	1.5 (2.5)
Blood and endocrine disorders	59 (11.3)	43 (12.7)	0.3 (0.5)	.03 (0.7)	10.4 (0.7)	0.7 (1.3)
Neuropsychiatric conditions	66 (12.7)	37 (10.9)	1.3 (2.1)	0.2 (4.8)	199.3 (13.1)	14.3 (24.5)
Sense organ diseases	11 (2.1)	10 (3.0)	0.0 (0.0)	0.0 (0.0)	86.9 (5.7)	4.4 (7.5)
Cardiovascular diseases	75 (14.4)	29 (8.6)	17.1 (29.0)	1.8 (42.0)	151.4 (9.9)	10.2 (17.4)
Respiratory diseases	11 (2.1)	6 (1.8)	4.0 (6.9)	0.2 (4.9)	59.0 (3.9)	3.1 (5.4)
Digestive diseases	10 (1.9)	4 (1.2)	2.0 (3.5)	0.2 (4.8)	42.5 (2.8)	2.7 (4.6)
Genitourinary diseases	23 (4.4)	13 (3.8)	0.9 (1.6)	.07 (1.6)	14.7 (1.0)	0.5 (0.8)
Skin diseases	7 (1.3)	7 (2.1)	.07 (0.1)	0.0 (0.2)	4.0 (0.3)	0.1 (0.2)
Musculoskeletal diseases	31 (6.0)	24 (7.1)	0.1 (0.2)	.02 (0.5)	30.9 (2.0)	2.6 (4.4)
Congenital anomalies	5 (1.0)	3 (0.9)	0.4 (0.7)	.01 (0.3)	25.3 (1.7)	0.6 (1.0)
Oral conditions	1 (0.2)	1 (0.3)	0.0 (0.0)	0.0 (0.0)	7.9 (0.5)	0.4 (0.7)

**Unintentional injuries **(poisoning)	**1 (0.2)**	**1 (0.3)**	**0.3 (0.6)**	**.01 (0.2)**	**7.5 (0.5)**	**0.2 (0.3)**

**Total**	**520 (100)**	**338 (100)**	**58.8 (100)**	**4.4 (100)**	**1,523.2 (100)**	**58.4 (100)**

Data do not sum to 100% because of rounding errors. Burden of disease data are from World Health Organization 2009 for the year 2004. Burden of disease is measured by mortality and disability-adjusted life years (DALYs). NCEs = New chemical entities. EU-25 = all 25 EU countries in 2004

**Figure 2:**
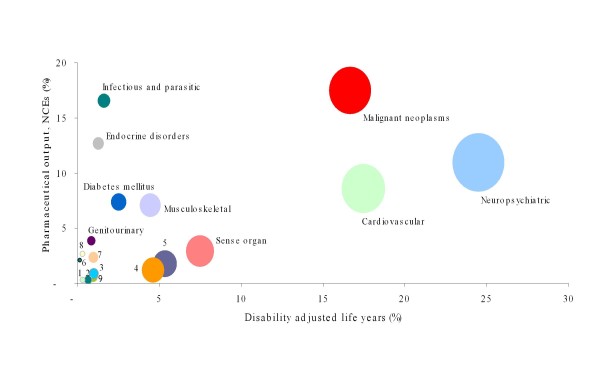
**Bubble plot representing disability-adjusted life years (DALYs) for EU-25 and active ingredients (NCEs)**. The areas of the bubbles are DALYs' weighted contribution of each disease condition(s) to the total burden of disease. 1: Other neoplasms; 2: Unintentional injuries (poisoning); 3: Congenital anomalies; 4: Digestive diseases; 5: Respiratory diseases; 6: Skin diseases; 7: Respiratory infections; 8: Maternal conditions; 9: Perinatal conditions.

**Figure 3:**
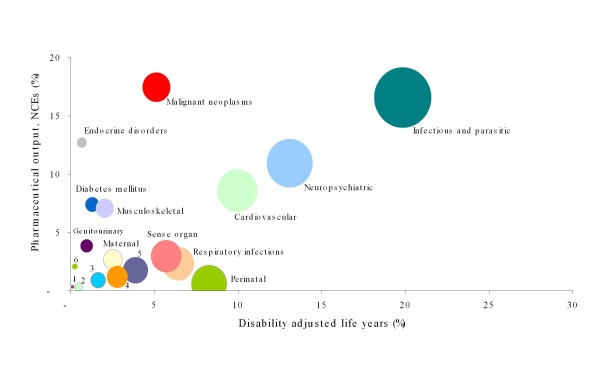
**Bubble plot representing disability-adjusted life years (DALYs) worldwide and active ingredients (NCEs)**. The areas of the bubbles are DALYs' weighted contribution of each disease condition(s) to the total burden of disease. 1: Other neoplasms; 2: Unintentional injuries (poisoning); 3: Congenital anomalies; 4: Digestive diseases; 5: Respiratory diseases; 6: Skin diseases.

In general, a high association was found between disease burden and the development of medicinal products in the EU-25 (ρ = 0.619, p = 0.005), and a moderate association at the global level (ρ = 0.490, p = 0.033) (Table 3). However, these associations do not hide disproportionality relationships between the various disease categories (Figures 2 and 3). In the EU-25, the greatest disproportionality was seen for infectious and parasitic diseases, blood and endocrine disorders, diabetes mellitus and genitourinary diseases (all of them overrepresented related to the burden they generate). Conversely, neuropsychiatric conditions, cardiovascular diseases, sense organ conditions, respiratory diseases, or digestive diseases were underrepresented (Figure 2). Meanwhile, the most neglected conditions worldwide (based on DALYs) were mainly perinatal conditions, respiratory infections, diseases of the sense organs, respiratory diseases, or digestive diseases (Figure 3).

**Table 3:** Associations of active ingredients (NCEs) with burden of disease (as measured by disability-adjusted life years and mortality) by region and country.

	Mortality		DALYs	
	Coefficient ρ	p-value	Coefficient ρ	p-value
**Worldwide**	**.417**	**.076**	.490*	**.033**

High-income	.485*	.035	.606**	.006
Low- and middle-income	.413	.079	.497*	.030

**EU-25**	**.449**	**.054**	.619**	**.005**
**EU-15**	.492*	**.032**	.603**	**.006**
Austria	.491*	.033	.619**	.005
Belgium	.554*	.014	.637**	.003
Denmark	.441	.059	.562**	.012
Finland	.334	.162	.376	.113
France	.555*	.014	.612**	.005
Germany	.455	.050	.657**	.002
Greece	.495*	.031	.610**	.006
Ireland	.484*	.036	.504*	.028
Italy	.455*	.558	.641**	.003
Luxembourg	.558*	.013	.585**	.008
Netherlands	.483*	.036	.534*	.018
Portugal	.476*	.039	.663**	.002
Spain	.518*	.023	.620**	.005
Sweden	.452	.052	.550*	.015
United Kingdom	.476*	.039	.564*	.012
**EU-10**	**.304**	**.206**	.499*	**.030**
Cyprus	.425	.070	.627**	.004
Czech Republic	.337	.159	.487*	.035
Estonia	.316	.188	.475*	.040
Hungary	.373	.116	.501*	.029
Latvia	.248	.306	.513*	.025
Lithuania	.207	.394	.404	.086
Malta	.328	.171	.578**	.010
Poland	.304	.206	.571*	.011
Slovakia	.326	.173	.547*	.015
Slovenia	.344	.149	.492*	.032

*p-value < .05 (bilateral); ** p-value < 0.01 (bilateral).

EU-25 = all 25 EU countries in 2004. EU-15 = established EU countries. EU-10 = 10 newly joined EU countries. Data represent Spearman's rank correlation coefficients (ρ). DALYs = disability-adjusted life years.

We found differences between regions and countries, with better associations with DALYs than with mortality (Table 3). By region, the associations were stronger in the EU-25 (DALYs: ρ = 0.619, p = 0.005) than in middle-low-income countries (DALYs: ρ = 0.497, p = 0.030) or globally (DALYs: ρ = 0.490, p = 0.033), and similar to those of high-income countries (DALYs: ρ = 0.606, p = 0.006). Differences also were found within the EU, with the associations being lower in the 10 countries recently joining the EU (DALYs: ρ = 0.499, p = 0.030) than in the 15 countries initially established (DALYs: ρ = 0.603, p = 0.006). By countries, the values were for mortality: from ρ = 0.304 (p = 0.206) in Poland to ρ = 0.558 (p = 0.013) in Luxembourg; while for DALYs, the values were from ρ = 0.376 (p = 0.113) in Finland to ρ = 0.663 (p = 0.002) in Portugal.

Additionally, we examined the pharmaceutical development output related to pediatric needs (Tables 4 and 5). We identified 103 PIPs agreed to in the EU after the Pediatric Regulation was introduced. No evidence of association was found between the number of PIPs and the regional burden of disease in children under 15 years (DALYs: ρ = 0.079, p = 0.779; mortality: ρ = 0.404, p = 0.135).

**Table 4:** Main characteristics of the Pediatric Investigation Plans (PIPs) and related active ingredients (NCEs)

Characteristics	Num. of PIPs (%)	Num. of NCEs (%)
**Total**	**103 (100)**	**95 (100)**

**Type of intervention**		
Prevention	11 (10.7)	8 (8.4)
Diagnosis	-	-
Treatment	85 (82.5)	80 (84.2)
Palliative care/rehabilitation	7 (6.8)	7 (7.4)

**Disease seriousness**		
Serious disease	73 (70.1)	66 (69.5)
Nonserious disease	20 (19.4)	19 (20.0)
Risk factor for serious disease	10 (9.7)	10 (10.5)

**New mechanism of action**	**21 (20.4)**	**20 (21.0)**

**Fixed-dose combinations**	**11 (10.7)**	**11 (11.6)**

**Biotechnological/biological product**	**31 (30.1)**	**25 (26.3)**

**Orphan medicinal product**	**16 (15.5)**	**16 (16.8)**

Data do not sum to 100% because of rounding errors. PIPs = Pediatric Investigation Plans. NCEs = New chemical entities with an agreed PIP. Source of the data: http://www.ema.europa.eu (search until September 2010).

**Table 5:** Distribution of pharmaceutical output related to pediatric needs by therapeutic area and burden of disease in children under 15 years (as measured by disability-adjusted life years and mortality)

Disease conditions	Pharmaceutical output related to pediatric needs		Burden of disease in children under 15 years			
	**PIPs**	**NCEs**	**Mortality per 10**^**6 **^**(%)**		**DALYs per 10**^**6 **^**(%)**	
	**Number (%)**	**Number (%)**	**Worldwide**	**EU-25**	**Worldwide**	**EU-25**

**Communicable diseases**	**20 (19.4)**	**19 (20.0)**	**10.1 (84.6)**	**0.1 (43.7)**	**406.5 (74.1)**	**0.9 (24.6)**
Infectious and parasitic diseases	12 (11.6)	12 (12.6)	4.6 (38.6)	.01 (3.4)	176.5 (32.2)	0.1 (3.8)
Respiratory infections	5 (4.8)	4 (4.2)	2.1 (17.3)	.01 (2.0)	76.2 (13.9)	.07 (1.9)
Maternal conditions	2 (1.9)	2 (2.1)	.00 (0.0)	.00 (0.0)	0.5 (0.1)	.00 (0.0)
Perinatal conditions	1 (1.0)	1 (1.0)	3.2 (26.8)	0.1 (38.3)	126.4 (23.1)	0.6 (16.2)

**Noncommunicable diseases**	**83 (80.6)**	**76 (80.0)**	**1.0 (8.1)**	**0.1 (44.3)**	**93.5 (17.1)**	**2.3 (64.2)**
Malignant neoplasms	16 (15.5)	15 (15.8)	.09 (0.7)	.02 (7.1)	3.2 (0.6)	.09 (2.4)
Other neoplasms	-	-	.01 (0.1)	.00 (0.9)	0.3 (0.1)	.01 (0.3)
Diabetes mellitus	9 (8.7)	7 (7.4)	.01 (0.1)	.00 (0.1)	0.3 (0.1)	.00 (0.1)
Blood and endocrine disorders	15 (14.6)	14 (14.7)	.05 (0.4)	.01 (3.2)	3.4 (0.6)	0.1 (3.8)
Neuropsychiatric conditions	9 (8.7)	9 (9.5)	.08 (0.6)	.02 (4.8)	30.6 (5.6)	0.9 (24.6)
Sense organ diseases	1 (1.0)	1 (1.0)	.00 (0.0)	.00 (0.0)	4.5 (0.8)	0.1 (3.6)
Cardiovascular diseases	12 (11.6)	11 (11.6)	0.1 (1.0)	.01 (2.7)	5.1 (0.9)	.04 (1.1)
Respiratory diseases	4 (3.9)	4 (4.2)	.06 (0.5)	.00 (1.1)	9.2 (1.7)	0.3 (9.0)
Digestive diseases	2 (1.9)	1 (1.0)	0.1 (1.1)	.00 (1.0)	6.5 (1.2)	.05 (1.7)
Genitourinary diseases	2 (1.9)	2 (2.1)	.03 (0.3)	.00 (0.2)	1.6 (0.3)	.01 (0.2)
Skin diseases	6 (5.8)	6 (6.3)	.00 (0.0)	.00 (0.0)	0.9 (0.2)	.00 (0.1)
Musculoskeletal diseases	7 (6.8)	6 (6.3)	.00 (0.0)	.00 (0.2)	1.6 (0.3)	.03 (0.9)
Congenital anomalies	-	-	0.4 (3.3)	.07 (23.1)	24.1 (4.4)	0.5 (14.5)
Oral conditions	-	-	.00 (0.0)	.00 (0.0)	2.2 (0.4)	.08 (2.2)

**Unintentional injuries **(poisoning)	-	-	**.03 (0.3)**	**.00 (0.2)**	**1.2 (0.2)**	**.00 (0.1)**

**Total**	**103 (100)**	**95 (100)**	**11.9 (100)**	**0.3 (100)**	**548.4 (100)**	**3.6 (100)**

Data do not sum to 100% because of rounding errors. Burden of disease data are from World Health Organization 2009 for the year 2004. Burden of disease in children under 15 years is measured by mortality and disability-adjusted life years (DALYs). PIPs = Pediatric Investigation Plans. EU-25 = all 25 EU countries in 2004. *NCEs = new chemical entities with an agreed PIP.

## Discussion

Our analysis for the period 1995-2009 uses the cohort of human-use medicinal products authorized by centralized procedure to show that the development of medicinal products is higher for some diseases than others. The three main therapeutic areas in terms of number of innovative medicinal products were oncology, infectious and parasitic diseases, and blood and endocrine disorders (accounting for 46.8% of active ingredients and 38.4% of marketing authorizations). The results also showed a moderate to high association between the development of medicinal products and disease burden measures for the main disease categories, though some conditions appear to be neglected (related to the health loss generated in the population) as in the case of neuropsychiatric, cardiovascular, respiratory, sense organ, digestive, perinatal diseases, etc. On the contrary, the number of agreed PIPs was not associated with the number of DALYs and deaths among children under 15 years. Therefore, the review of PIPs suggested again that some disease conditions were more neglected than others (related to pediatric health needs), such as perinatal conditions, congenital anomalies, and neuropsychiatric diseases.

The European Medicines Agency is the regulatory authority providing the Member States and the institutions of the EU with scientific advice on quality, safety, and efficacy of medicines. Medicinal products authorized by the centralized procedure can be automatically marketed in all EU Member States. Barbui and Garattini [12] highlighted some issues that can adversely affect assessment of medicinal products for some diseases. First, the centralized procedure is not mandatory for all human-use medicinal products, and different authorization procedures in the EU (e.g., centralized versus mutual recognition/decentralized and national procedures) can result in heterogeneity in terms of authorized medicinal products between countries. Second, new medicinal products can be evaluated without needing to establish comparisons to active treatments. Therefore, a medicinal product can be marketed if it shows a difference versus a placebo (dummy treatment), and in cases where this is not ethical, demonstrating "noninferiority" versus an active comparator is permitted. This can cause uncertainty on the therapeutic role of new medicinal products, as suggested in recent reviews on cancer [13,14], cardiovascular [15], antirheumatic [16], and central nervous system treatments [17]. From a public health perspective [18-20], the therapeutic value and degree of innovation of medicinal products could also be considered, referring to their added value related to other available options. Some of the items to be highlighted in this approach would include: the relative or incremental value compared with the available alternatives, the evaluation under real conditions of use (comparative effectiveness and safety), or even its incremental costs (efficiency or cost-effectiveness), which is of high value for guiding pricing and reimbursement decisions following the marketing authorization [21,22].

While there is general concern about the lack of innovation in the development of medicinal products, there is an increased number of active ingredients and of new marketing authorizations. The apparent increase in centrally authorized generics, "biosimilars," and fixed-dose combinations observed over the study period may have contributed to this situation. Furthermore, these results may be a sign of the failure of the pharmaceutical market to anticipate unmet needs and consumer demand.

This analysis also involves some limitations. First, the assessment refers only to medicinal products for human use authorized by centralized procedure. Though there are other alternative procedures, we assumed that the cohort of medicinal products selected here can be considered to be representative of current pharmaceutical innovation. Second, to measure pharmaceutical development output, we used for the primary analyses the data on new marketing authorizations and of active ingredients. Despite being significant measures expressing the productivity of research and development activities, other measures could have been selected instead (e.g., funding for pharmaceutical research, total expenditures per therapeutic area or per DALY lost, etc.). The number of new marketing authorizations and of active ingredients may not be necessarily an indication of the interest in a given disease as they may represent drugs that are structurally similar to already existing ones with only minor pharmaceutical differences (so-called "me-too" drugs). In addition, broader health status measures favorably reflect the results of biomedical research investments. These include lower DALYs lost, lower death rates for chronic diseases, longer life expectancy and improved quality of life for elderly people; and more effective and earlier disease detection [23]. Third, sometimes a medicinal product can be authorized for more than one therapeutic indication in various conditions or diseases (e.g., an anticancer agent for the treatment of solid tumors and hematological malignancies, a psychoanaleptic indicated for depression and diabetic peripheral neuropathic pain, etc.). For the purpose of our analysis, the information can be imperfect as the main indication was chosen. However, we think using the main disease groups and categories instead of subcategories while also using a comprehensive, consistent, and exclusive classification system enabled us to reduce misclassification bias, making the main findings robust.

The approach of this analysis has been used in other areas, such as health services research [24-26]. Surprisingly, little attention has been paid to analyzing disproportionality among population health needs and the development of innovative medicinal products. The parties interested in some diseases or interventions often mention the lack of funds for their disease or intervention without considering the global health implications. A previous report concluded that health research does not reflect the global burden of disease, with less than 10% of resources invested in the study of diseases that contribute 90% of the global burden of disease, also known as the "10/90 gap" [27]. It has been argued that the pharmaceutical industry appears unwilling to fund development of new medicinal products aimed at health needs in middle-low-income countries because the prospects for financial gain for the industry are limited [28]. Our analysis provides information on this matter. We found that nearly 80% (262/338) of the new medicinal products are aimed at less than 50% of health losses (expressed in DALYs) in the world, accounting for 90% of DALYs in high-income countries, as in the case of the EU-25. Although the data appear to indicate that the "10/90 gap" is decreasing, there are still neglected global health needs.

It is noteworthy to mention that the availability of medicines depends partially on the state of research on a particular disease. Indeed, some diseases may require a more significant investment of resources to develop innovative medicines. The lack of novel medicinal products in specific areas may also indicate that there are medicines under patent protection (marketing exclusivity) or that the pharmaceutical market is saturated in those areas. Therefore, the financial rewards for the pharmaceutical industry to develop medicines of relevant therapeutic value may not be profitable enough to assume the risky nature of their investments.

On the other hand, the granting of marketing approval does not necessarily translate into improved availability of medicines. In fact, access to medicines is far from being globally harmonized because price and reimbursement are still matters for countries to consider according to their government policies, health resources, and public health systems [14]. This is particularly important in middle-low-income countries, where there is an access problem either for issues linked to the cost of medicines acquisition, defective health structures, or underdiagnosis. For example, about 5 million people die every year worldwide because of communicable diseases such as diarrheal conditions, HIV/AIDS, tuberculosis, and malaria [10], even though these diseases are treatable (or at least preventable) with current interventions. In the case of noncommunicable diseases such as diabetes or other cardiovascular risk factors (e.g., hypertension, obesity, smoking, etc.) for which interventions (not only medicines) are available, many health systems cannot meet population needs in the poorest communities. Chronic noncommunicable diseases in developing countries are not just diseases of the elderly, since cardiovascular diseases account for as many deaths in young and middle-aged adults as HIV/AIDS. Also, chronic diseases affect a much higher proportion of people in developing countries during their prime working years than they do in developed countries [29]. There are several challenges to public health that require a new global and regional strategic approach, including the availability of medicines for current unmet medical needs, among others [30,31]. Some investigators have suggested incentives for new medicinal products that are effective against diseases of high societal burden or gravity for populations. These include incentives for novelty, comparative effectiveness and safety, extended patent life, and pricing enhancements for drugs aimed at particular public health needs. Such incentives have been used successfully for vaccines and low-prevalence (orphan) diseases [29].

The European Medicines Agency has recently launched a public consultation on its future actions, based on which it intends to set priorities for coming years. European and international agencies, including medical and patient organizations, the pharmaceutical industry, and citizens have been invited to participate in the document, "Road Map to 2015" [31], including debating how to approach public health needs, promoting research on medicinal products in unmet medical needs areas, or for rare and neglected diseases, providing innovative proposals for drug development.

In this paper, we intended to provide information based on the outcomes of decisions made during 15 years in the European setting. Pharmaceutical industry leaders and policymakers are invited to consider the implications of the imbalance explored in this paper, establishing work plans that allow for defining future priorities from a public health perspective.

## Competing interests

The authors declare that they have no competing interests.

## Authors' contributions

FCL conceived the study aims and design, and developed the study in discussions with AGA, EAM, RGM, and CMG. FCL performed the analysis and drafted the initial manuscript. All authors contributed to interpretation of results, revised and commented on the manuscript for important intellectual content, and approved the final version.
